# Methyl linolenate suppresses the growth and proliferation of Ehrlich ascites carcinoma (EAC) cells by inducing intrinsic mitochondrial apoptosis

**DOI:** 10.17179/excli2025-8865

**Published:** 2026-01-05

**Authors:** Azmin Akter, Tasnima Kamal, M. Matakabbir Hossain, Abdul Auwal, Khan Mohammad Rashel, Tasfik Ul Haque Pronoy, Asmaulhusna Biswas, Sharmin Akter, Mahmud Ismail, M. Rowshanul Habib, Farhadul Islam

**Affiliations:** 1 Department of Biochemistry and Molecular Biology, University of Rajshahi, Rajshahi-6205, Bangladesh; 2 School of Medicine and Dentistry, Griffith University, Gold Coast Campus, Queensland-4222, Australia

**Keywords:** Clerodendrum viscosum, methyl linolenate, phytomedicine, tumor weight, survival, angiogenesis, apoptosis

## Abstract

A new bioactive compound, methyl linolenate (Methyl-octadeca-9,12,15-trienote), designated as ML, was isolated and purified from *Clerodendrum viscosum* leaves. Treatment of Ehrlich ascites carcinoma (EAC) cells with ML induced cancer growth inhibition dose-dependently, with a maximum cell growth inhibition of 67 % at a dose of 3.0 mg/kg/day (p<0.001). It also inhibits EAC cell volume and tumor weight and increases the survival time of EAC-bearing mice (25 versus 41 days) (p<0.001). In addition, EAC-bearing control mice exhibited a drastic deterioration of blood parameters, and treatment of EAC-bearing mice with ML prevented the deterioration of hematological parameters compared to untreated EAC-bearing mice. Also, ML abrogates angiogenesis by inhibiting the development of new blood vessels in the peritoneum of EAC-bearing mice. ML-treated cells exhibited apoptotic features such as condensed, fragmented nuclear material and cell membrane damage. Expression of pro-apoptotic genes such as *p53, Bax, Caspase 3, and Caspase 9* was upregulated, whereas anti-apoptotic gene *Bcl2* was downregulated in ML-treated EAC cells, which indicates the induction of intrinsic mitochondrial apoptosis of EAC cells. However, as it is a novel anticancer compound showing an antineoplastic effect, inhibiting angiogenesis, and inducing apoptosis in mouse models, thus, using other cellular models and more preclinical and clinical research is essential for further development.

See also the graphical abstract(Fig. 1).

## Introduction

Cancer, a collective term for the uncontrolled proliferation of cells and a wide range of disorders characterized by aberrant cellular function, is the second leading cause of mortality globally (Kikuchi et al., 2019[26]; Shendge et al., 2017[45]). There are several methods of treatment for patients with cancer, including chemotherapy, radiation therapy, surgery, immunotherapy, endocrine therapy, and gene therapy etc. Among the treatment options, chemotherapy is the most common method and widely used in clinical settings (Tilsed et al., 2022[49]; Kikuchi et al., 2019[26]). Chemotherapeutics kill rapidly proliferating cancer cells, inducing apoptosis of cancer cells; however, they also damage normal cells, resulting in a high incidence of complications (Yuan et al., 2017[54]). In addition, the efficacy of conventional chemotherapeutics has been limited by drug resistance (Housman et al., 2014[15]). Thus, the limited efficacy, being nonspecific to cancer cells, affects normal healthy tissues, and adverse effects are considered a major hindrance to the effective use of chemotherapy in patient management (Yousefi Rizi et al., 2022[53]). Therefore, the development of therapeutics, especially those based on natural products, to overcome the limitations of chemotherapy is urgently needed.

Since ancient times, medicinal plants have been utilized in complementary, alternative, and traditional systems for the ailment of various pathophysiological conditions, including cancers (Shukla and Mehta, 2015[46]). Nature supplies an endless number of molecules with novel mechanisms of action and distinctive structural models, and many of the substances, such as bleomycin, paclitaxel, docetaxel, etc., have been reported to be used in cancer treatments (Floyd et al., 2005[12]). In fact, they can be used in a versatile manner, including as chemotherapeutic agents, chemopreventive agents, or chemosensitizers to increase the efficacy of traditional chemotherapies (Ruiz-Torres et al., 2017[42]; Singh et al., 2016[48]; Calcabrini et al., 2017[7]; Kotecha et al., 2016[27]; Wang et al., 2012[51]; Vinod et al., 2013[50]). The natural products, especially the secondary metabolites from plants with anti-cancer properties, caused the induction of apoptosis of cancer cells, inhibited proliferation, migration, angiogenesis, and altered cell cycle kinetics (Choudhari et al., 2020[9]; Islam et al., 2014[17]; Siddika et al., 2021[47]). Furthermore, natural plant-based products overcome the acquired chemoresistance of cancer cells and minimize the toxic side effects of anticancer medications, thereby enhancing the cytotoxic efficiency of chemotherapy by sensitizing tumor cells (de Oliveira Júnior et al., 2018[10]).

*Clerodendrum viscosum *(Family: Lamiaceae, common name: bhant, ghentu), also known as *Clerodendrum infortunatum*, is a weed of agricultural fields, is widely distributed throughout Africa, Australia, America, Asia, and the Indian subcontinent (Nandi and Lyndem, 2016[33]). It is a hill glory bower plant that has been used extensively in Ayurvedic medicine for ages. For example, it has been documented that *C. viscosum* can treat a number of diseases such as skin conditions, intestinal infections, kidney dysfunction, snake bites, scorpion stings, etc (Prashith Kekuda et al., 2019[37]; Wang et al., 2017[52]). The entire plant juice is used to treat worm infections, fever, bronchitis, leprosy, coughing, itching, scorpion stings, and other ailments (Roy et al., 2022[41]). The methanolic extract of this plant's leaves has been shown to have a bundle of bioactivities, including antioxidants, antimicrobial, antihelmintic, hepatoprotective, anti-inflammatory, antinociceptive, hypoglycemic, wound healing, antidiarrheal, and sedative activities (Bhattacharjee et al., 2011[5]). In addition, several phytochemicals, such as flavonoids and phenolic compounds, show anticancer activities by inhibiting cancer cell proliferation, inducing apoptosis, and causing cell cycle arrest. For example, apigenin and acacetin, identified from *C. viscosum*, exhibited remarkable anticancer effects, including ROS generation, apoptosis initiation, and G2/M-phase cell cycle arrest (Nandi and Lyndem, 2016[33]).

In this study, we purified a bioactive compound, named methyl linolenate (ML), from *C. viscosum* leaves for the first time from this plant. Previous investigations revealed that ML from vegetable oils is able to produce ROS-mediated antifungal properties against *Paracoccidioides brasiliensis* (Pinto et al., 2017[36]). Also, ML from *Celtis australis* fruits exhibited antibacterial activity (Badoni et al., 2010[3]). ML from *Oxalis triangularis* demonstrated stronger anti-melanogenic effects in cultured mouse B16 melanoma cells by reducing cAMP production and inhibiting tyrosinase promoter activity, which are critical for melanin biosynthesis (Huh et al., 2010[16]). Therefore, it's indicated that this compound could demonstrate antiproliferative properties against other cancer cells. Thus, the traditional uses of *C. viscosum* leaves and the existing literature on the rich pharmacological properties of bioactive compound ML led us to hypothesize that ML from *C. viscosum* leaves could be a potential candidate for an effective chemotherapeutic agent. Hence, the current study focuses on the purification and characterization of ML and the assessment of antineoplastic effects of this compound against EAC cells using* in vivo* assays. This study also demonstrates the possible mechanism through which ML suppresses tumor cell growth and proliferation.

## Materials and Methods

### Materials

The leaves of the *C. viscosum* plant were collected from the vicinity of Rajshahi University campus during November and December in 2023. The identification of the plant was carried out by Dr. Md. Rezaul Karim, a professor in the Department of Botany, Rajshahi University, Rajshahi, Bangladesh. A voucher specimen has been preserved in the Department of Botany at Rajshahi University, Bangladesh.

### Extraction and purification

The extraction and isolation procedures were carried out according to a previously established protocol (Islam et al., 2015[19]). Disease-free, mature leaves were collected, washed, and minced into tiny pieces, and then dried in moderate sunlight. Then, a grinding tool (FFC-15, China) is used to break the material into a coarse powder. The powder (about 500 g) was put in a separate, clean, round-bottomed glass bottle with 2 L of 95 % methanol. The container's contents were sealed with aluminum foil and a cotton plug, and it was kept for 10 days with occasional shaking and stirring. The final extracts were filtered using Whatman no. 1 filter paper, and the solvents were then evaporated at 40° C at lower pressure in a rotatory evaporator, yielding a 20-gram crude methanol extract. The extract was then subjected to various chromatographic techniques, including thin-layer chromatography (TLC), column chromatography (CC), and preparative thin-layer chromatography (PTLC) for separation and purification. For TLC, the extract was dissolved in methanol and applied to TLC plates, which were developed using a mobile phase of n-hexane/ethyl acetate (7:3). The plates were dried and examined under ultraviolet light at wavelengths of 254 and 366 nm. A specific active band was scraped off from each plate. To visualize the bands, vanillin-sulfuric acid reagent was also applied. Then, column chromatography techniques were used to separate the compound into different fractions in a sufficient amount. For this experiment, an extract (~2 g) was applied on a 0.15-0.3 mm silica gel (Merck, Germany) and chromatographed using n-hexane with a gradient of low-polarity solvent system to high-polarity solvent system up to 100 % ethyl acetate. A total of 285 fractions were collected, and fractions 141-200 were further subjected to preparative thin-layer chromatography, which gave a single spot. The purified compound was obtained in oily form (~50 mg), and the compound was characterized by spectrometric analysis.

### Spectrometric analysis

Spectrometric analysis was performed to determine the structure of the purified compound. Nuclear magnetic resonance (NMR) spectra were recorded on a 400 MHz FT spectrometer DPX-400 (Burker, Karlsruhe, Germany). The analysis was performed in the Analytical Research Division, Wazed Miah Science Research Centre (WMSRC), Jahangirnagar University, Savar, Dhaka-1342, Bangladesh.

### Experimental animal and ethical approval of the study

The use of experimental animals (mice) and protocols of the current study was approved by the Institutional Animal, Medical Ethics, Biosafety, and Biosecurity Committee for the experiments involving animals, humans, microbes, and living natural sources. The protocols and approved guidelines are strictly followed throughout the study. In the current study, male adult Swiss albino mice, weighing 20-25 g, were recruited from our animal facility, where the animals were meticulously raised and reared according to the standard protocols and regulations as approved (No. 293(13)/320-IAMEBBC/IBSc). Also, the study complied with the ARRIVE guidelines and the policies and laws of the National Institutes of Health Office of Laboratory Animal Welfare. The animals were maintained in controlled, hygienic conditions, housed in standard polypropylene cages, and under a 12 h light/12 h dark cycle at a temperature of 24±2 ℃. Drinking water and standard food were given *ad libitum*. The highest animal welfare considerations, such as efforts for minimal distress and suffering of mice, were taken by monitoring the behaviours and health of the mice twice a day (morning and evening). For anesthesia, the animals were put in a chamber (30 cm × 30 cm) at the end of the experiment. Then, a 5 % Isoflurane (1.2 L/minute flow rate) in oxygen for two to four minutes was given to induce anesthesia. Breathing pattern and anesthesia depth were monitored by regular toe pinching and ensure an adequate sedated state of the mice without being over anesthesized. Subsequently, mice were taken on a surgical table, and 1-2 % Isoflurane (1.2 L/minutes flow rate) in oxygen was given to maintain anesthesia. The animals were sacrificed by the cervical dislocation method, and humane endpoint was ensured for them, and efforts were taken to minimize animal suffering.

### Cell line

In this study, Ehrlich Ascites Carcinoma (EAC), recognized as a transplantable tumor, was used. The EAC cells were provided by the Indian Institute of Chemical Biology (IICB) in Kolkata, India. These cells were maintained in an animal through intraperitoneal transplantation in Swiss albino mice.

### Cell growth inhibition

The effect of the purified compound on EAC cell growth was examined by a previously published method (Siddika et al., 2021[47]; Lampronti et al., 2003[28]). In short, four groups of Swiss albino mice, six mice in each group, weighing 25-30 g, were used. 1 × 10^6^ EAC cells were introduced into each group of mice on day zero. Treatment commenced 24 hours post-tumor inoculation and persisted for five days. Group 1 was used as a control, EAC cell-bearing mice without treatment. Groups 2 and 3 received test compounds at the doses of 0.3 and 3.0 mg/kg/day, respectively, via intraperitoneal injection, whereas group 4 received the standard anticancer drug ifosfamide at the doses of 0.4 mg/kg/day. Mice of each group were sacrificed on day 6, and the total intraperitoneal cancer cells were harvested with 0.98 % normal saline. Viable cells were first identified with trypan blue and then counted by a hemocytometer under an inverted microscope (XDS-1R, Optika, Bergamo, Italy).

### Determination of the survival time and average tumor weight

The assessment of the survival time of EAC-bearing mice, as well as the tumor weight, was carried out using the methods that have been previously reported (Khanam et al., 2010[25]). Three groups (n=6) of Swiss albino mice were used. For therapeutic evaluation, 1×10^6^ EAC cells per mouse were inoculated into each group of mice on day 0. Treatment started after 24 hours of tumor inoculation and continued for 10 days. Group 1 was used as a control group of EAC cell-bearing mice without treatment. Groups 2 and 3 received test compounds at the doses of 0.3 and 3.0 mg/kg/day, respectively, via intraperitoneal injection. The tumor growth of the mice was monitored by recording the daily weight change. The survival time was recorded and expressed as mean survival time (MST) in days, and the percentage increase of life span (%ILS) was calculated as follows:

MST = (Σ Survival time in days of each mouse in a group) / Total number of mice

%ILS = (MST of treated group / MST of the control group - 1) x 100

### Monitoring hematological parameters

Hematological profiles of the experimental mice were evaluated to assess the effects of the test compound on the blood parameters of EAC-bearing mice as previously described (Islam et al., 2014[21]). In this experiment, four groups (n=6) of mice were used. Groups 1, 2, and 3 received EAC cells (0.1 mL of 1.6×10^6^ cells/mice) intraperitoneally at day zero, whereas group 4 consisted of normal mice without treatment. After 24 hours of EAC inoculation, groups 2 and 3 were treated with the test compound at 0.3 and 3.0 mg/kg body weight of mice/day, respectively. Group 1 was used as a control, EAC cell-bearing mice without treatment. On the 12th day, hematological parameters (hemoglobin, RBC, and WBC) were measured from the freely flowing tail vein blood of each mouse of each group and compared with EAC-bearing control and healthy untreated animals.

### Morphological changes and nuclear damage

The morphological characteristics of apoptotic nuclei were assessed by fluorescence microscopy using propidium iodide, as previously described (Banfalvi, 2017[4]). To evaluate whether the reduction in cell growth due to the test compound is attributed to apoptosis, an analysis of nuclear morphology was conducted with the aid of the fluorescent DNA-binding agent propidium iodide. The morphological characteristics of EACs in both the treated group (3.0 mg/kg/day) and the control group were analyzed using a fluorescence microscope. The cells (500 μl) were centrifuged at 1200 rpm, discarded from the supernatant, and subsequently rinsed with PBS buffer (1 ml), and stained with propidium iodide of 10 μl for 15 minutes in a dark place. The nuclear morphology of apoptotic cells, characterized by condensed or fragmented nuclei, was analyzed under a fluorescent microscope.

### Peritoneal angiogenesis

To examine the impact of the test compound on peritoneal angiogenesis, the animals' abdominal cavities were opened after they had been sacrificed, and the inner lining of the peritoneal cavity was examined to identify new blood vessel formation as previously published protocol (Ashwini et al., 2023[2]). Subsequently, photographs were captured of the peritoneal cavities from both control mice carrying EAC cells and treated mice for comparison.

### mRNA extraction from EAC cells and cDNA synthesis from mRNA

Total RNA was extracted using FavorPrep^TM^ Blood/ Cultured Cell Total RNA Purification Mini Kit (Favorgen Biotech Corp., Ping Tung 908126, TAIWAN) from EAC cells. Nanodrop one (Thermo Scientific) was used to determine the concentration and quality of the RNA, with the mean absorption ratios A260/280 and A260/230 evaluated to ensure purity. RNA integrity was assessed using 1.8 % agarose gel electrophoresis. The cDNA synthesis reaction was carried out using the High-Capacity GoScript^TM^ Reverse transcription system following the manufacturer's guidelines.

### Real-time quantitative PCR (RT-qPCR)

To examine the expression of genes followed by compound treatment in EAC cells, RT-qPCR was used. Expression of growth-regulating genes such as *p53, Bax, Bcl2, Caspase 3, *and* Caspase 9 *was investigated, with GAPDH as the loading control. In brief, a 20 μL reaction mixture was created with 2x Universal SYBR Green Fast qPCR Mix, 25 pmol of both forward and reverse primer, and 50 ng of cDNA. A LightCycler 96 PCR (ROCHE, 4414 Lake Boone Trail, Raleigh, NC 27607, USA) was used. The PCR amplification conditions consisted of 10 min at 95° C followed by 45 cycles of a denaturation step at 95° C for 15 seconds and annealing and extension for 1 min at 60° C. These data were analyzed using the comparative Ct (ΔΔCt) method. The relative expression level of a gene was calculated by determining a ratio between the amount of the gene and that of endogenous control glyceraldehyde-3-phosphate dehydrogenase (GAPDH) as previously described. The fold change was calculated using the 2^(-ΔΔCt) method (Islam et al., 2018[18]). The list of primers used in the current study is given in Supplementary information (Supplementary Table 1).

### Statistical analysis

The percentage of cell growth inhibition, an increase in animal life span, and tumor weight were measured in six identical copies for each experiment, and the mean ± SEM (standard error of the mean) was expressed based on the findings. Using GraphPad Prism 8 software, a one-way ANOVA and Duncan's multiple range test were used to analyze the data. A value was deemed significant when it was p<0.05.

## Results

### Purification and spectrometric analysis of the compound

**Figure 2:**
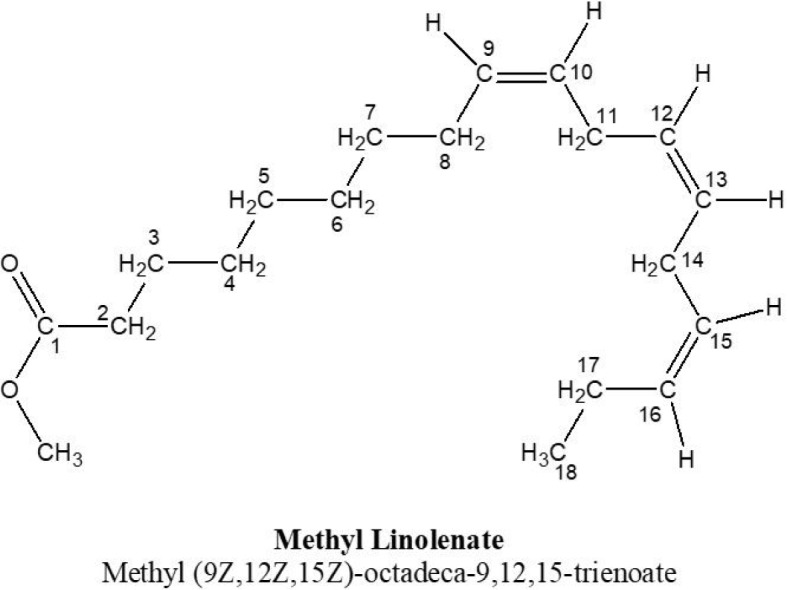
Structure of the purified compound. The structure of the compound methyl linolenate (ML) [Methyl (9Z,12Z,15Z)-octadeca-9,12,15-trienoate] was determined by NMR spectrometric data analysis.

The purified compound was obtained as a colorless oil and characterized by NMR spectra data. The ^1^H-NMR spectrum of compound exhibited six olefinic proton at 5.43 ~ 5.28 (2H, m, H-9, H-10, H-12; H-13, H-15, H-16) thereby ensuring the presence of three double bonds. Its ^1^H-NMR spectrum demonstrated the presence of three methylene (-CH_2_ -) proton signal as triplet at 2.31 (2H, t, J = 7.5 Hz) for H-2 and at 2.83 ~ 2.79 (2H, t, J = 5.6 Hz) for H-11 and H-14. In addition, signals with multiple splitting patterns were also observed at 1.63 ~1.60, 1.35 ~ 1.30, and 2.14 ~ 2.08 for seven methylene protons in a ^1^H-NMR spectrum of compound-1. Moreover, two signals assigned to methyl protons were found at 1.01 ~ 0.97 (triplet) and 3.33 (singlet). The ^13^C-NMR spectrum of compound-1 showed an acetyl carbonyl carbon at δ 176.3 and six olefinic carbons at δ 131.3 (C-9), 129.6 (C-10), 127.8 (C-12), 127.7 (C-13), 127.4 (C-15), and 126.8 (C-16). Based on all the above evidence and comparison with reported data **(**Huh et al., 2010[16]), the structure of the compound was deduced as methyl linolenate [Methyl (9Z,12Z,15Z)-octadeca-9,12,15-trienoate] (Figure 2(Fig. 2)).

### Properties of compound-1 (i.e., Methyl Linolenate)

Colorless oil; ^1^H-NMR (400 MHz, CD_3_OD, δ, ppm, *J*/Hz): 2.31 (2H, t, *J* = 7.5 Hz; H-2), 1.63 ~1.60 (2H, m, H-3), 1.35 ~ 1.30 (2H, m, H-4), 1.35 ~ 1.30 (2H, m, H-5, H-6, H-7), 2.14 ~ 2.08 (2H, m, H-8), 5.43 ~ 5.28 (2H, m, H-9, H-10, H-12; H-13, H-15, H-16), 2.83 ~ 2.79 (2H, t, *J* = 5.6 Hz, H-11, H-14), 2.14 ~ 2.08 (2H, m, H-17), 1.01 ~ 0.97 (3H, t, *J* = 7.6 Hz, H-18), 3.33 (3H, s, -OCH_3_).

^13^C-NMR (400 MHz, CD_3_OD, δ, ppm): 176.3 (C-1), 33.5 (C-2), 25.1 (C-3), 29.3 (C-4), 29.2 (C-5), 29.2 (C-6), 29.2 (C-7), 29.1 (C-8), 131.3 (C-9), 129.6 (C-10), 26.7 (C-11), 127.8 (C-12), 127.7 (C-13), 25.1 (C-14), 127.4 (C-15), 126.8 (C-16), 20.0 (C-17), 13.2 (C-18), 48.1 (-O-CH_3_). The spectra were presented in the Supplementary information (Supplementary Figures 1-7). The compound has a molecular formula of C_19_H_32_O_2_ with an exact mass is 292.25. Its molecular weight is recorded as 292.46. The mass-by-charge ratio (m/e) is observed at 292.24 (100.0 %), 293.24 (21.2 %), and 294.25 (2.2 %). The percentage of element composition is as follows: carbon (C)=78.03 %, hydrogen(H)=11.03 %, and Oxygen(O)=10.94 %.

### Purified methyl linolenate induces (ML) EAC cell growth inhibition in vivo

**Figure 3:**
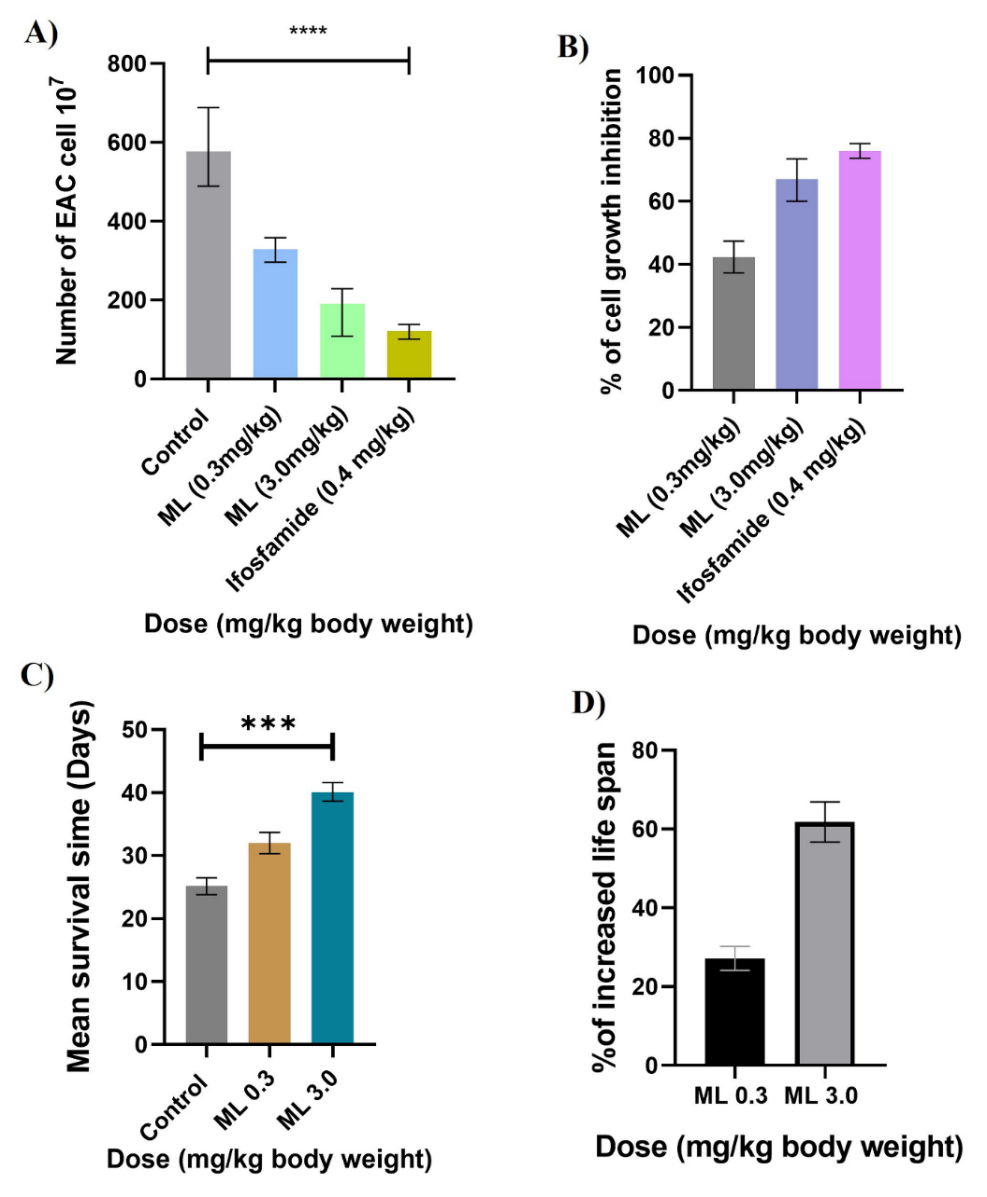
Effect of ML on EAC cell growth and survival of EAC-bearing mice. A) Number of viable EAC cells in control and ML-treated mice. The values represent the mean ± SEM (n=6). B) Percentage of cell growth inhibition at different doses compared to control mice. C) Effect of ML on mean survival time of EAC-bearing mice (n=6) compared to untreated EAC-bearing mice (n=6). D) The percentage of life span increases with the treatment of experimental mice (n=6) compared to EAC-bearing control mice (n=6). The results are represented as the mean ± SEM from independent experiments (n=6). Level of significance ****p<0.001 and ***p<0.01 when compared with the control group by Duncan's multiple range test.

The effect of methyl linolenate (ML) and ifosfamide (standard drug) on the growth of EAC cells is illustrated in Figures 3(Fig. 3) (A and B). Treatment with ML resulted in a significant reduction of viable EAC cells (Figure 3A(Fig. 3)). The highest inhibition of cell growth recorded was 67.03 % at a dosage of 3.0 mg/kg body weight relative to the control (p<0.001). Concurrently, the mice treated with the standard anticancer drug ifosfamide (0.4 mg/kg) demonstrated a 75.95 % inhibition of cell growth in comparison to that of the control (Figure 3B(Fig. 3)).

### ML increased the survival time and life span of EAC-bearing mice

The effect of ML on the mean survival time and percent increase of life span at different doses is shown in Figures 3C & 3D(Fig. 3), respectively. ML at 3.0mg/kg body weight treatment caused an increase (58.76 %) in life span, significantly compared to that of the control mice (p<0.001). Whereas a 24.7 % life span increment was noted in EAC-bearing mice receiving ML (0.3 mg/kg) in comparison to that of the control.

### Reduction of tumor burden in ML-treated EAC-bearing mice

**Figure 4:**
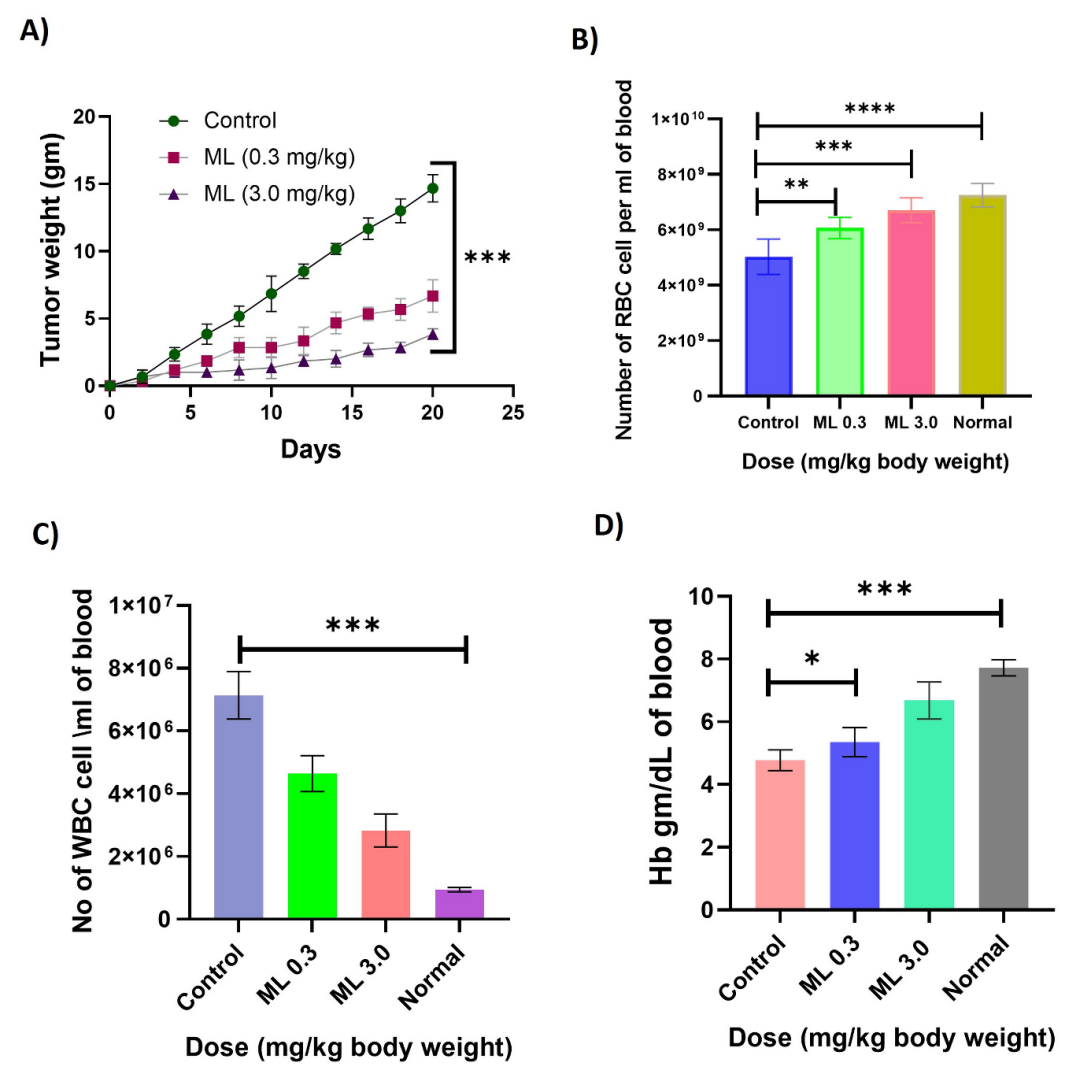
Effect of ML on tumor weight and blood parameters of EAC-bearing mice. A) Reduction of tumor burden of EAC-bearing mice (n=6) at different doses of ML. B) Effect of ML on RBC on EAC-bearing control (n=6) and treated mice (n=6). C) Effect of ML on WBC counts on treated and non-treated EAC-bearing mice. D) Level of Hb (gm/dl) of mice receiving ML and untreated control mice. Data are expressed as mean ± SEM (n=6) from independent experiments. Level of significance ****p<0.001, ***p<0.01, and **p<0.05 when compared with the control group by Duncan's multiple range test.

It has been observed that treatment with the test compound on mice previously inoculated with EAC cells decreased the tumor weight (Figure 4A(Fig. 4)). In the case of the control (EAC bearing) group, the tumor weight was increased when compared to the normal. In mice treated with ML at doses of 3.0 mg/kg/day, the tumor weight was decreased. It was noted that on day 20, the tumor burden of ML-treated EAC-bearing mice was significantly reduced (14.66 gm versus 3.83 and 6.66 gm, respectively, at 3.0 and 0.3 mg/kg body weight) in comparison to control EAC-bearing mice (Figure 4A(Fig. 4)).

### Altered hematological parameters in methyl linolenate-treated mice

Hematological parameters were found to have deteriorated from normal values along with the growth of the tumor. After tumor inoculation, the number of RBC, WBC, and % of Hb were drastically changed. However, the altered hematological parameters were restored towards normal levels in treated mice at the dose of 3.0 mg/kg body weight (Figure 4B-D(Fig. 4)).

### Methyl linolenate inhibits angiogenesis in EAC-bearing mice

**Figure 5:**
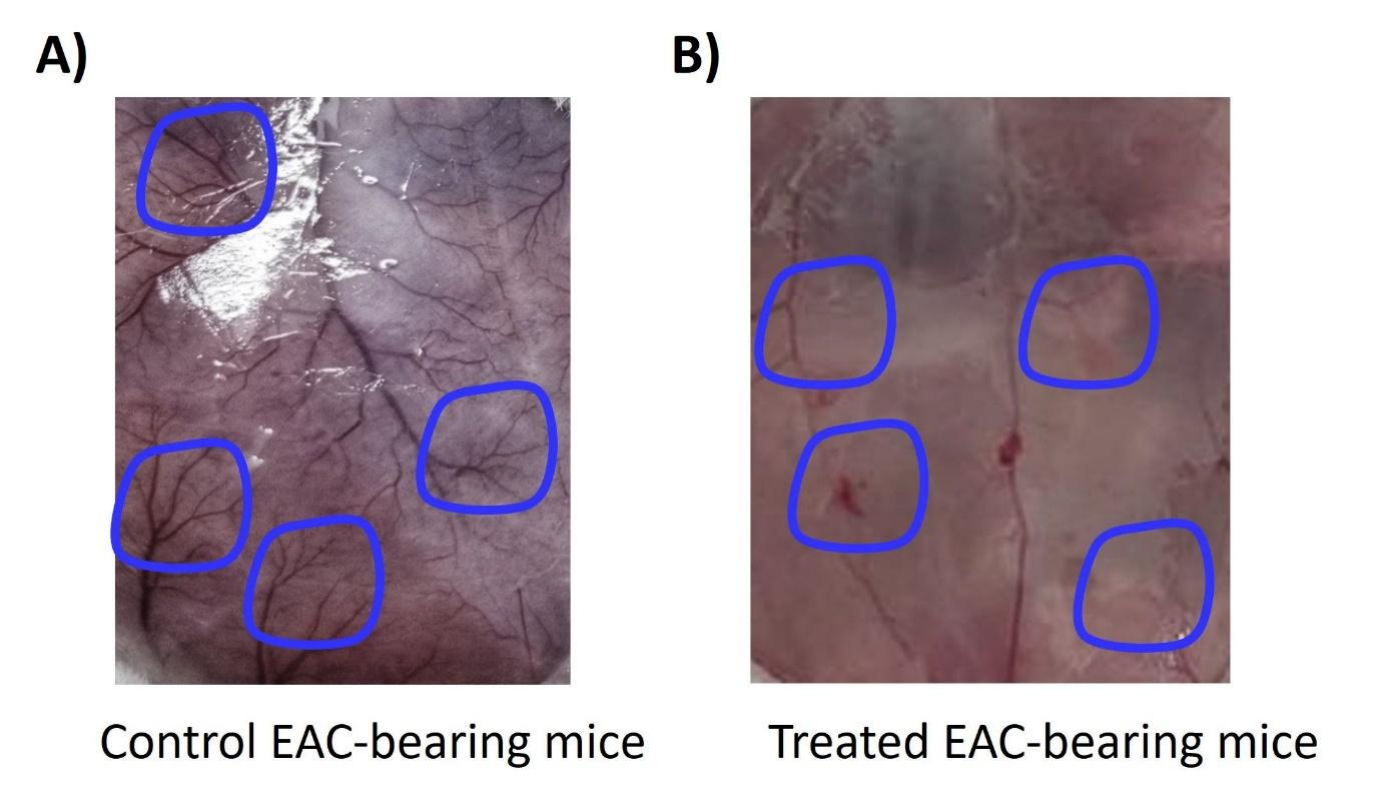
Effect of ML on peritoneal angiogenesis. A) Extensive neovascularization is observed in the peritoneal lining of EAC-bearing control mice (Blue circled) (n=6). B) Remarkable inhibition of peritoneal angiogenesis is evident in ML-treated EAC-bearing mice (Blue circled) (n=6). Representative images from the independent experiments (n=6).

The effect of ML on the development of new blood vessels in the peritoneum of EAC-bearing mice is shown in Figure 5(Fig. 5). It was noted that ML strongly inhibited peritoneal neovascularization when compared to the control mice's peritoneal cavity.

### ML-induced morphological changes and nuclear damage of EAC cells

**Figure 6:**
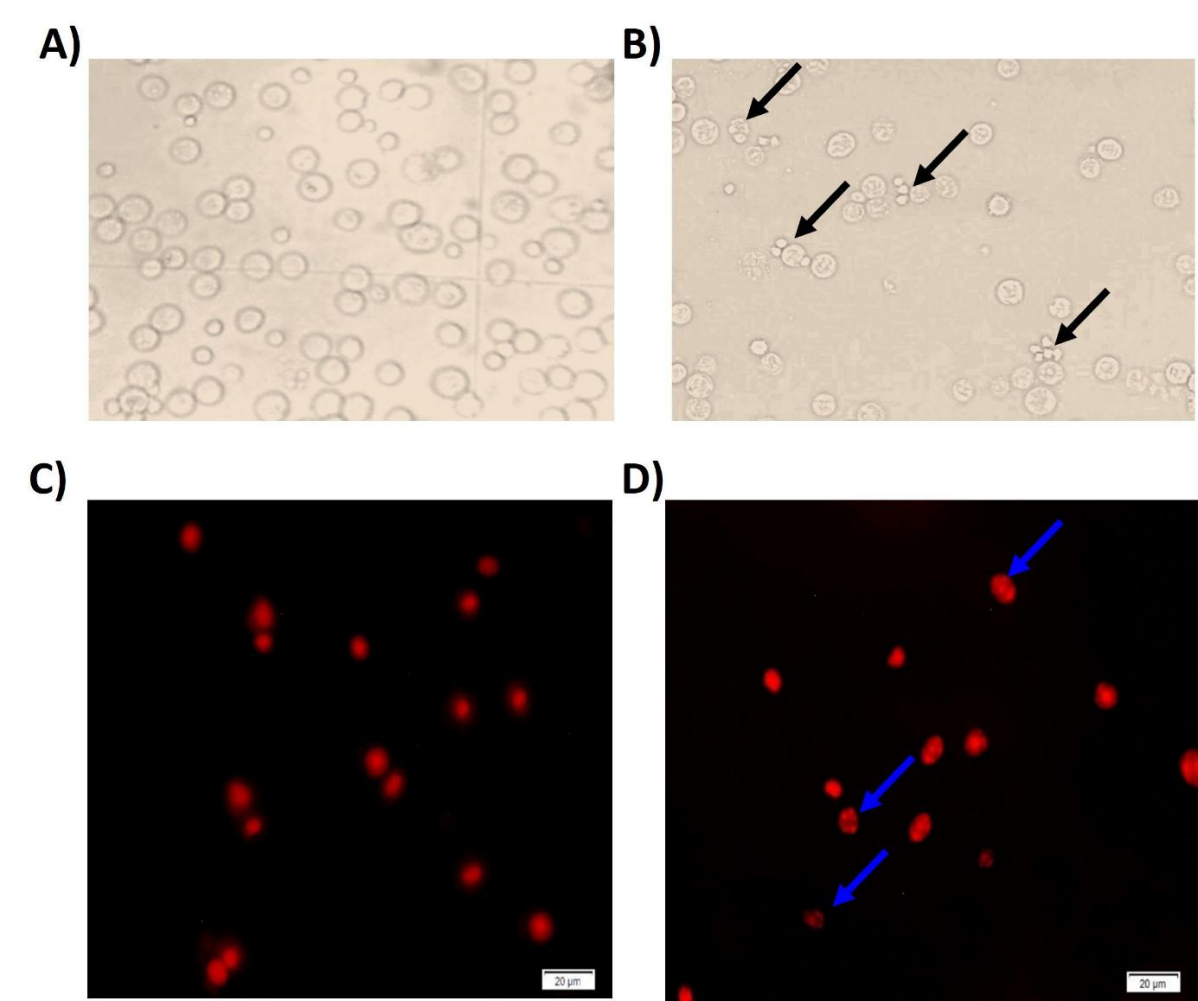
Morphological changes of EAC cells. Light microscopy indicated that the control group had healthier and more cells (A) in comparison to those of ML-treated cells (B). C) Control EAC cells under a fluorescence microscope after propidium iodide staining. D) The treatment with ML resulted in the apoptosis of EAC cells by the formation of apoptotic bodies (indicated by the arrows). Cells were obtained from EAC-bearing mice (n=6) that had not received treatment and those that had received the treatment (n=6), followed by staining with propidium iodide and observation through fluorescence microscopy. Representative images from the independent experiments (n=6).

Morphological changes of EAC cells followed by ML treatment are shown in Figure 6(Fig. 6). The nuclei of EAC cells in the control group exhibited a round, regular shape (Figure 6A(Fig. 6)) and were uniformly stained with propidium bromide, as illustrated in Figure 6(C)(Fig. 6). In contrast, EAC cells treated with ML displayed evident fragmentation of DNA within their nuclei, as depicted in Figure 6(B & D)(Fig. 6). Thus, ML markedly elevated the number of apoptotic cells in comparison to the control group, as demonstrated by the presence of nuclear fragmentation and condensation (Figure 6(Fig. 6)).

### Upregulation of pro-apoptotic and downregulation of anti-apoptotic genes in ML-treated EAC cells

**Figure 7:**
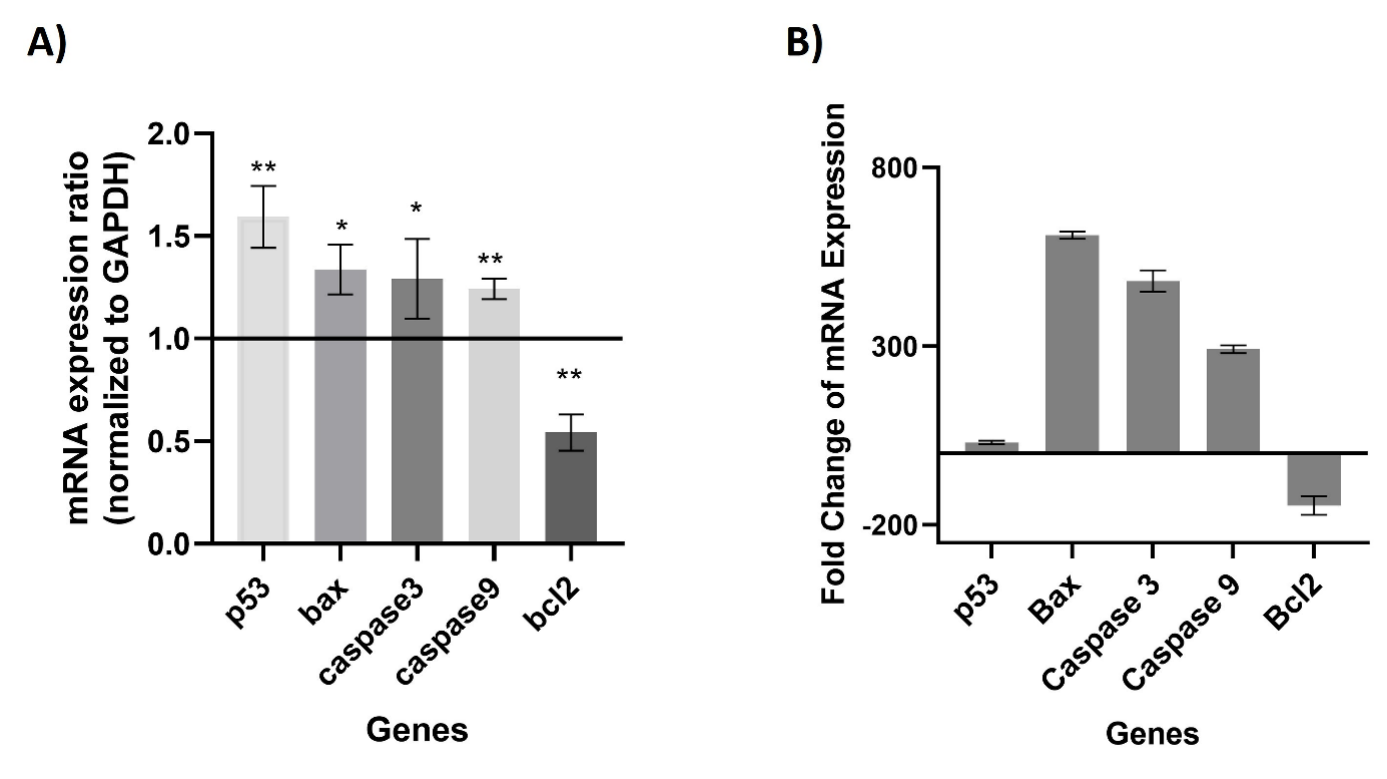
Overexpression of apoptosis-related genes in ML-treated EAC cells. A) Relative expression of *p53, Bax, Caspase 3, Caspase 9*, and *Bcl2* in EAC cells treated with the compound and followed by normalization to the untreated control cells. Proapoptotic genes such as *p53*, *Bax*, *Caspase 3*, and *Caspase 9* were upregulated while anti-apoptotic gene *Bcl2* down downregulated in cells receiving ML treatment in comparison to untreated control cells. Data was analyzed by ΔΔ CTs and normalized to the housekeeping gene GAPDH. (2way ANOVA test p<0.05). B) Expression changes in fold changes of the tested genes in treated cells. The fold change was calculated using the 2^(-ΔΔCt) method. All results are represented as the mean ± SEM from independent experiments (n=6) with triplicate technical replication. Level of significance **p<0.01, and *p<0.05 when compared with the control group by Duncan's multiple range test.

The expression of apoptotic-related genes was studied using RT-qPCR from total RNA extracted from ML-treated and untreated EAC cells. It was noted that the mRNA expression level of pro-apoptotic genes such as *p53, Bax, Caspase 3, *and* Caspase 9 *was significantly upregulated in ML-treated cells (Figure 7(Fig. 7)). On the other hand, expression of anti-apoptotic gene *Bcl2* was significantly downregulated in ML-treated EAC cells (Figure 7(Fig. 7)).

## Discussion

The current study analyzes *C. viscosum* leaf extract to explore the anticancer compounds of the plant, as different parts of the plant have many bioactive compounds with various pharmacological activities. Herein, we have purified and isolated a compound, namely methyl linolenate (ML), from the leaves for the first time in this plant. Then, we examined the anticancer activity of the purified ML against EAC cells by monitoring several parameters such as cell growth inhibition, the survival time of tumor-bearing animals, tumor weight reduction, and hematological parameters. In addition, the possible mechanism of anticancer activity of ML was investigated using microscopic and gene expression analysis. The purified compound, ML, exhibited dose-dependent anticancer activity against EAC cells by inducing the intrinsic apoptotic pathway.

The phytochemical composition of the methanolic extract from the leaves of *C. viscosum* includes reducing sugars, tannins, triterpenoids, steroids, saponins, flavonoids, and alkaloids (Penu et al., 2022[35])*.* GC-MS analysis of leaves methanolic extract reported the presence of nineteen distinct bioactive compounds such as methyl pyruvate dimethyl acetal, o-anisic acid, 3-chloroprop-2-enyl ester, tridecane, silane, ethyl dimethyl phenyl, pentadecane, dodecane, 4,6-dimethyl, phenol, 4-(1-methylpropyl), azacyclohexane, 3-methylamino-1-methyl, dodecane, 2, 6, 11-trimethyl, heptadecane, octadecane, 1-iodo, phenol, 3,5-bis(1, 1-dimethylethyl), methyl 6, 6, 8, 8-tetramethyl-3oxo-2,5,7,9-tetra, hexadecanoic acid, methyl ester, phytol, retionic acid, 2-propenoic acid, 2-methyl-1,2-ethanediylbis, 4a(2H)-Naphthalenol, 2-bromo-4,4-dichloro, 1-cyclohexanol, 2-[1-(phenylsulfonyl)methyl (Ghosh et al., 2015[13]). Thus, the isolated and purified ML can be considered a novel compound from *C. viscosum* leaves identified in the current study. However, ML has been purified from leaves of *T. angulata var. intermedia* (Han et al., 2021[14]). Also, ML was purified from the ethanolic extract of the whole bodies of *Oxalis triangularis* (Huh et al., 2010[16]). Methyl gamma linolenate was isolated from the *Spirulina platensis* by flash chromatography system, which exhibited potent cytotoxic activity towards human lung carcinoma cells (Jubie et al., 2015[23]). Nevertheless, there is no report regarding the anticancer activity of ML obtained from *C. viscosum* leaves against EAC cells.

Plant-derived bioactive products have been reported to exhibit potent anti-tumor activity against several animal and human cancer cell lines through inducing apoptosis and cell death (Lawal and Özaslan, 2021[29]). For example, paclitaxel (trade name Taxol), a natural clinically used anticancer drug, is a tricyclic diterpenoid produced in the bark and needles of *Taxus brevifolia* has been used in patients with various cancers in clinical settings (Zhu and Chen, 2019[56])*. *Also, p-menth-1-ene-4,7-diol (EC-1) isolated from *Eucalyptus camaldulensis Dhnh.* showed EAC cell growth (73 %) inhibition at a dose of 2 mg/kg (Islam et al., 2015[19]. Similarly, our compound ML could effectively inhibit a cell growth rate of 67 % at a dose of 3 mg/kg. The prolongation of animal lifespan is a critical parameter for determining the effectiveness and viability of antitumor drugs. Treatment with a pure compound, deoxyelephantopin (DOE) from *Elephantopus scaber,* resulted in a significant increase in both the mean survival time and the percentage of the lifespan of mice compared to those in the control group (Kabeer et al., 2019[24]). Accordingly, treatment with ML has been demonstrated to enhance the survival time of EAC-bearing mice, increasing the mean survival time from 25 days to 41 days. An antitumor antibiotic, bleomycin (0.3 mg/kg), isolated from *Streptomyces verticellus,* reduced tumor weight by 21 % in 20 days compared to control EAC-bearing mice (Morshed et al., 2011[32]). Similarly, ML has shown a notable decrease in tumor weight in EAC-bearing mice in the current study (14.66 versus 3.83 g). The hematological parameters, such as RBC, WBC, hemoglobin (Hb), and platelet counts, were altered from normal values in mice afflicted with cancer, whereas treatment restored the parameters to normal values (Islam et al., 2012[20])**. **Bleomycin treatment significantly enhanced the numbers of RBC, hemoglobin, and lymphocytes with the corresponding depletion of the WBC, neutrophils, and monocytes in the experimental mice (Sharmin et al., 2008[44]). According to the previous study, treatment with the ML reversed all the depleted hematological parameters towards normal. These findings elucidate the potential of ML and may contribute to the development of more effective phytotherapeutics aimed at better management of patients with cancers.

A hallmark of tumor growth and proliferation is increased angiogenesis, which facilitates the delivery of nutrients and oxygen and removes waste products from metabolism, enabling uncontrolled cell division and ultimately metastasis (De Palma et al., 2017[11]). Therefore, preventing increased angiogenesis is a crucial therapeutic approach for slowing the growth of tumors. It was noted that our test compound ML has been shown to impede peritoneal angiogenesis, which is a vital event for tumor growth and progression. This compound possesses potent antiangiogenic properties by suppressing the formation of neo-vasculature in the peritoneum of EAC-bearing mice.

**Figure 8:**
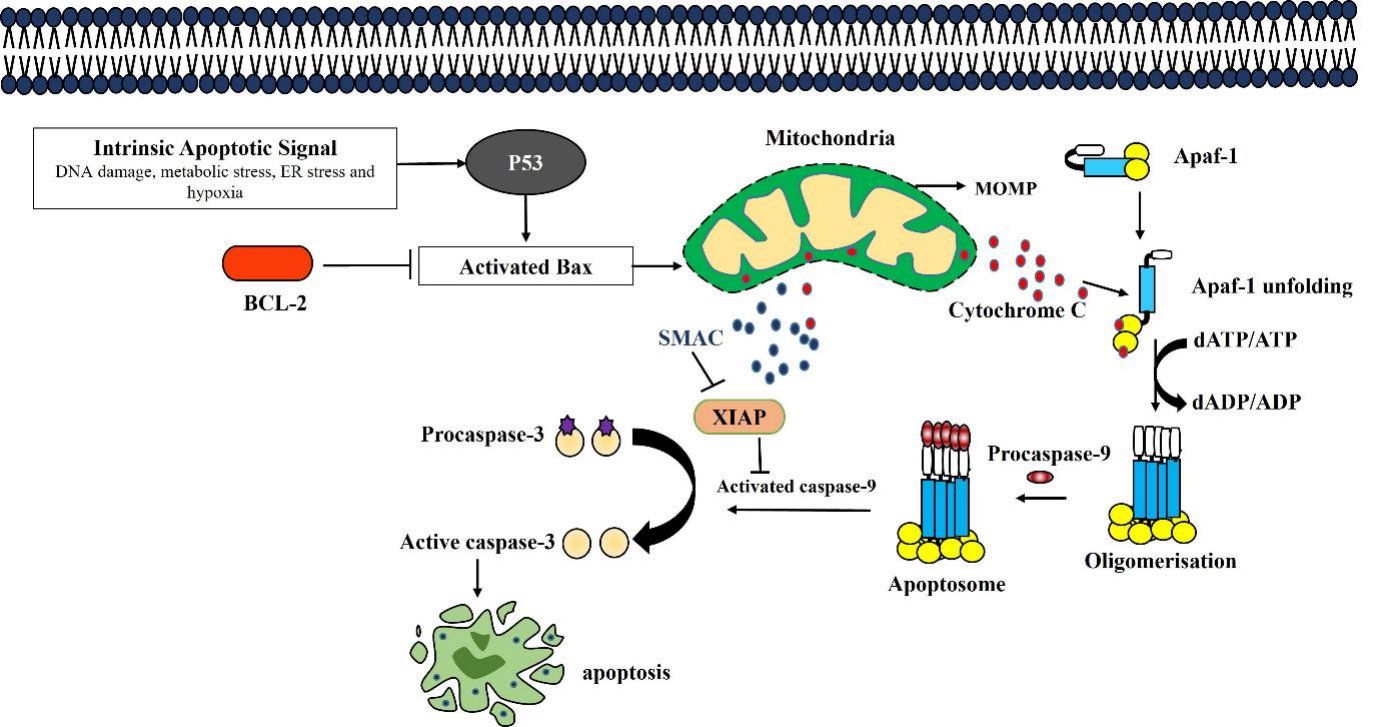
Possible targeting apoptotic pathway induced by ML in EAC cells. Apoptosis can be executed via the intrinsic mitochondrial pathway. In this process, the majority of stimuli trigger apoptosis through the mitochondrial pathway, with mitochondrial outer membrane permeabilization (MOMP) being the critical event. Following MOMP, proteins from the mitochondrial intermembrane space, particularly cytochrome c, are released into the cytosol, where they initiate the activation of caspases. Cytochrome C is pivotal in the creation of the apoptosome, a complex that activates Caspase 9 and Caspase 3, while Smac alleviates the suppression of Caspases by neutralizing inhibitors of apoptosis (IAP) proteins. APAF (Apoptotic Protease Activating Factor), BAX (Bcl2 Associated X Protein), XIAP (X-linked Inhibitor of Apoptosis Protein), Smac (Second Mitochondria-derived Activator of Caspase), Bcl2 (B-cell Leukemias and Lymphomas).

Phenotypically, apoptosis is marked by distinct features such as the fragmentation of DNA, shrinkage of the cell, condensation of chromatin, the occurrence of blebbing on the plasma membrane, and the eventual collapse of the cell into small, intact fragments termed apoptotic bodies (Alam et al., 2016[1]). We have noted that ML treatment triggered morphological changes in EAC cells, including DNA fragmentation, cell shrinkage, and DNA condensation, which are consistent with previous reports, suggesting that ML may induce apoptosis in EAC cells. To confirm the nature of cell death, we performed RT-qPCR to analyze the apoptotic gene expression. It was also reported that several plant-derived bioactive compounds induce apoptosis by activating intrinsic pathways of apoptosis (Siddika et al., 2021[47]; Nurujjaman et al., 2024[34]). Interestingly, apoptotic induction has been identified as a promising target for innovative mechanism-based drug discovery (Sankari et al., 2012[43]). Numerous anticancer medications have been developed through a screening process that identifies compounds from plants. Notable examples include vinblastine and vincristine sourced from *Catharanthus roseus*, Taxol derived from *Taxus brevifolia*, etoposide and teniposide obtained from *Podophyllum *species, as well as topotecan and irinotecan from *Camptotheca acuminata*, which induce apoptosis of highly proliferating cells. Additionally, 4-ipomeanol is extracted from *Ipomoea batatas*, and ß-lapachone is derived from *Tabebuia avellanedae* showed anticancer activity against various cancers. These plant-based drugs are known to promote apoptosis in various cancer types (Rahman et al., 2021[39]). Proapoptotic and antiapoptotic genes are differentially regulated during apoptosis. In the process of cancer formation, proapoptotic genes are downregulated and anti-apoptotic gene expression is increased, allowing cells to evade apoptosis and develop into cancer (Miah et al., 2020[31]). Several naphthoquinonoid natural products with antitumor properties, e.g., shikonin, lapachol, β-lapachone, diospyrin, etc., have been reported as inducers of apoptosis in human cancer cells by regulating distinct genes such as *p53, Bcl2, caspases,* etc. (Chakrabarty et al., 2002[8]). Magnolol, a natural compound, triggers mitochondrial apoptosis pathway of SGC-7901 human gastric adenocarcinoma cells by an increased ratio of Bax/Bcl-2, dissipation of mitochondrial membrane potential, and sequential activation of caspase 3 and inhibition of PI3K/Akt signaling pathways (Rasul et al., 2012[40]). Similarly, we examined the mRNA expression of many apoptotic genes, such as *p53, Bax, Caspase 3, caspase 9*, and* Bcl-2 *in EAC cells of both treated and untreated groups. There was a downward trend in antiapoptotic genes such as *Bcl-2* expression, while there was an upward trend in pro-apoptotic genes such as *p53, Bax, Caspase 3, and caspase 9* following ML treatment. These findings suggest the activation of mitochondrial intrinsic apoptosis induction of EAC cells (Figure 8(Fig. 8)).

Phytochemicals, including flavonoids, alkaloids, and glycosides, are implicated in the upregulation of the *p53* gene, which is a key modulator of apoptosis following DNA damage and cell cycle arrest (Islam et al., 2018[22]). In this study, ML upregulates *p53* expression in EAC cells. The upregulation of proapoptotic *Bax* and downregulation of anti-apoptotic *Bcl-2* genes act as the key players in the intrinsic pathway of apoptosis (Rahi et al., 2020[38]). The *Bcl-2* family of proteins plays a crucial role in regulating the mitochondrial pathway of apoptosis by managing the permeability of the outer mitochondrial membrane. In response to DNA damage, *p53* binds *Bcl-2* family members, *BH_3_*-only proteins, thus activating the pro-apoptotic gene Bax to transmit an apoptotic signal to the mitochondria that ultimately leads to cell death (Brunelle and Letai, 2009[6]). The permeabilization of the outer mitochondrial membrane, which facilitates the release of cytochrome C and cytochrome C plays a crucial role in establishing a platform for the activation of Caspase 9 and Caspase 3, known as the apoptosome (Huh et al., 2010[16])*.* The activation of Caspase 3 is responsible for the characteristic features of apoptosis, such as DNA fragmentation and cell death in various tissues (Lopez and Tait, 2015[30])*.* The test compound, ML, upregulates the expression of Caspase 3 and Caspase 9, which indicates the induction of apoptosis.

In recent years, plant-derived anticancer agents have garnered considerable attention because of their reduced toxic effects (Zhou et al., 2021[55]). Thus, it can be anticipated that ML will demonstrate a reduced toxic effect, which will be the subject of further investigation. In addition, the long-term toxicity and side effects should be explored in further study. This research is also limited by the low yield of purified ML from the crude extract. However, synthetic and semi-synthetic chemical moieties could be a source of alternative sources of compounds.

The results altogether indicate that the purified compound, ML, derived from the leaves of *C. viscosum* possesses notable potential as an anticancer agent. It exhibits anticancer properties by promoting apoptosis and protecting the host's hematological profile, thereby presenting itself as a promising option for cancer therapy (Figure 8(Fig. 8)). The intrinsic mitochondrial pathway of apoptosis may serve as a promising target for novel therapeutic interventions, and effectively targeting this pathway may have the potential to revolutionize cancer treatment options. This study may pave the way for the development of a new, cost-effective natural drug for cancer treatment. However, the outcomes of the present research do not provide a definitive basis to declare an accomplishment in the formulation of a new anticancer medication. Before making such an assumption, it's important to conduct comprehensive, sophisticated research projects using several cell lines as well as higher animal models, such as human cell lines, to prove it as an innovative cancer treatment drug.

## Declaration

### Conflict of interest

The authors declare that they have no conflict of interest.

### Artificial Intelligence (AI) - assisted technology

We did not use any artificial intelligence in this manuscript.

### Data availability

Data and information presented in the manuscript will be available upon request to the corresponding author of the manuscript.

### Funding declaration

This project was supported by a grant from the Faculty of Science, Rajshahi University, Rajshahi-6205, Bangladesh (265/5/52/RU/Science-35/2023-2024).

### Author contribution

Azmin Akter: Data analysis and writing the draft, Tasnima Kamal, M. Matakabbir Hossain, Abdul Auwal: Data curation, writing original draft preparation. Khan Mohammad Rashel, Tasfik Ul Haque Pronoy, Asmaulhusna Biswas, Sharmin Akhter, Mahmud Ismail: Data analysis: M. Rowshanul Habib: Spectral data analysis, Farhadul Islam: Writing- Reviewing and Editing, Supervision.

## Supplementary Material

Supplementary information

## Figures and Tables

**Figure 1:**
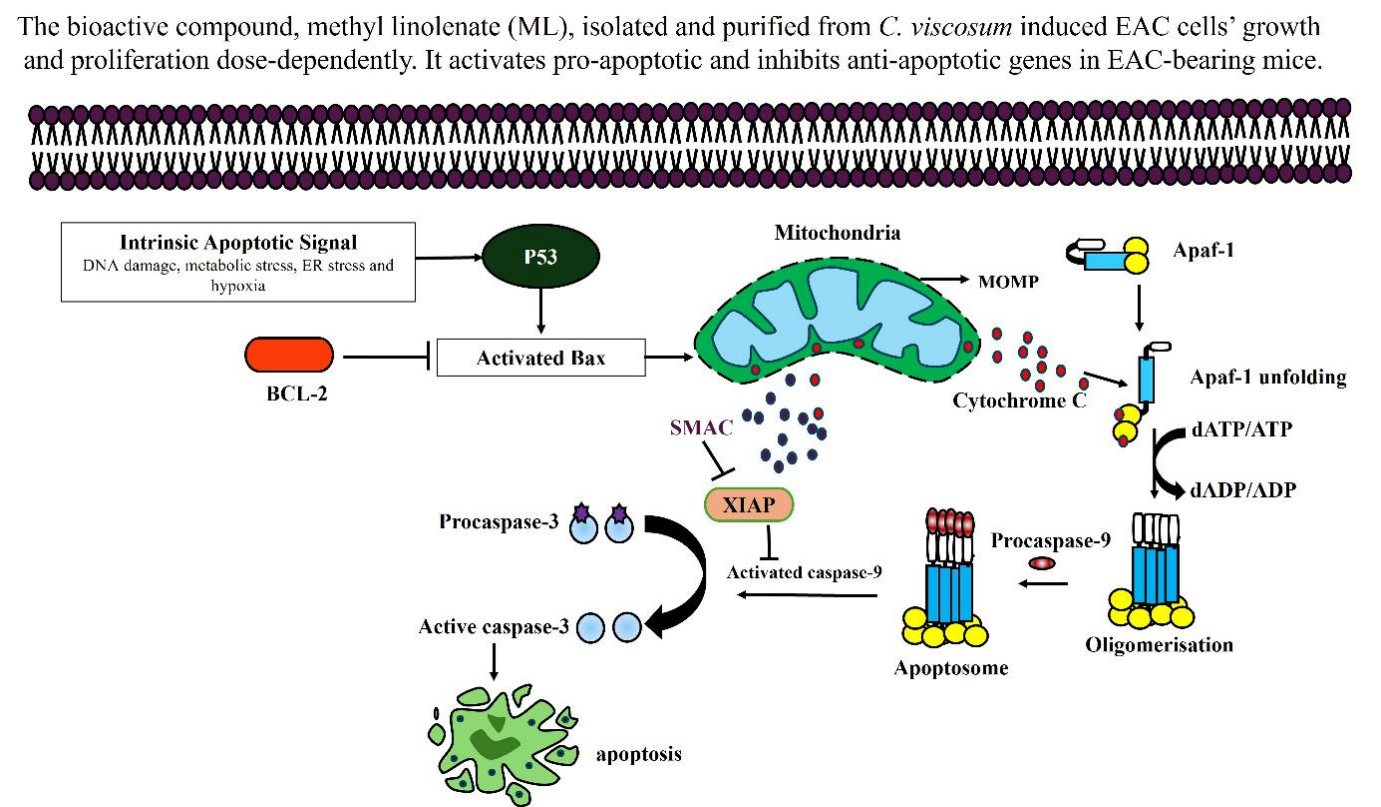
Graphical abstract
