# Supplementary material for: Methyl linolenate suppresses the growth and proliferation of Ehrlich ascites carcinoma (EAC) cells by inducing intrinsic mitochondrial apoptosis
Source: EXCLI J. 2026 Jan 5;25:50–67. doi: 10.17179/excli2025-8865 (PMC12901954; doi:10.17179/excli2025-8865)
Supplement: Supplementary information [file EXCLI-25-50-s-001.pdf]

EXCLI Journal 2026;25:50-67 – ISSN 1611-2156
Received: August 26, 2025, accepted: October 13, 2025, published: January 05, 2026

Supplementary information to:

Original article:

METHYL LINOLENATE SUPPRESSES THE GROWTH AND PROLIFERATION OF EHRLICH ASCITES CARCINOMA (EAC) CELLS BY INDUCING INTRINSIC MITOCHONDRIAL APOPTOSIS

Azmin Akter[1], Tasnima Kamal[1], M. Matakabbir Hossain[1], Abdul Auwal[1], Khan Mohammad Rashel[1], Tasfik Ul Haque Pronoy[1], Asmaulhusna Biswas[1], Sharmin Akter[1], Mahmud Ismail[1], M. Rowshanul Habib[1], Farhadul Islam[1,2*]

[1] Department of Biochemistry and Molecular Biology, University of Rajshahi, Rajshahi-6205, Bangladesh
[2] School of Medicine and Dentistry, Griffith University, Gold Coast Campus, Queensland-4222, Australia

* **Corresponding author:** Farhadul Islam, Department of Biochemistry and Molecular Biology, University of Rajshahi, Rajshahi-6205, Bangladesh;
E-mail: farhad_bio83@ru.ac.bd;
School of Medicine and Dentistry, Griffith University, Gold Coast Campus, Queensland-4222, Australia; E-mail: f.islam@griffith.edu.au

https://dx.doi.org/10.17179/excli2025-8865

This is an Open Access article distributed under the terms of the Creative Commons Attribution License (https://creativecommons.org/licenses/by/4.0/).

Supplementary Table 1: List of primers used for gene expression analysis

Gene name	Primer sequence	Generating band
GAPDH	Forward: (5'-GTGGAAGGACTCATGACCACAG-3')<br>Reverse: (3'-CTGGTGCTCAGTGTAGCCCAG-5')	0.475 kb
P^{53}	Forward: (5'-GCGTCTTAGAGACAGTTGCCT-3')<br>Reverse: (3'-GGATAGGTCGGCGGTTCATGC-5')	0.458kb
Bax	Forward: (5'-GGCCCACCAGCTCTGAGCAGA-3')<br>Reverse:(3'-GCCACGTGGGCGTCCCAAAGT-5')	0.479 kb
Bcl-2	Forward: (5'-GTGGAGGAGCTCTTCAGGGA-3')<br>Reverse: (3'-AGGCACCCAGGGTGATGCAA-5')	0.304 kb
Caspase-3	Forward: (5'-TGG ACTGTGGCATTGAGACAG-3')<br>Reverse: (3'- CGACCCGTCCTTTGAATTTC-5')	0.137 kb
Caspase-9	Forward: (5′-GCTGCCTGAGAAGTACAAAGA-3′)<br>Reverse: (3′-TCGTTGGGCAGGTTCTTATC-5′)	0.487 kb

EXCLI Journal 2026;25:50-67 – ISSN 1611-2156
Received: August 26, 2025, accepted: October 13, 2025, published: January 05, 2026

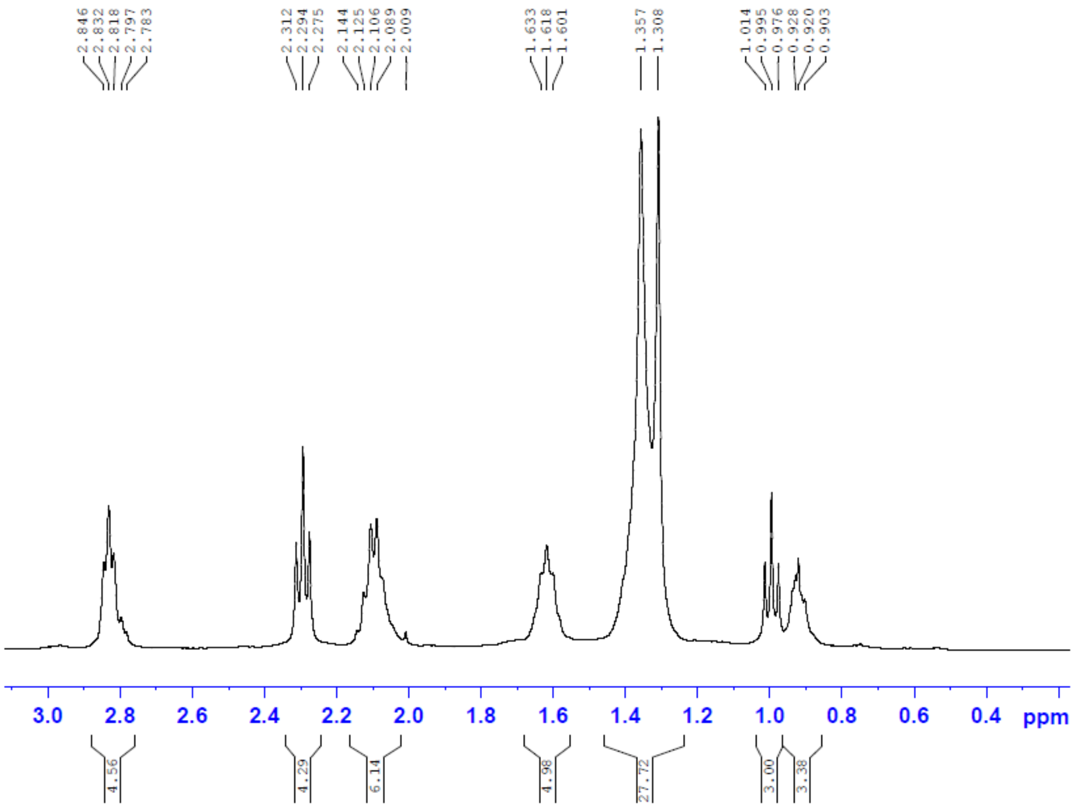

Supplementary Figure 1: ^{1}H NMR Spectral data (part-1) for the purified compound

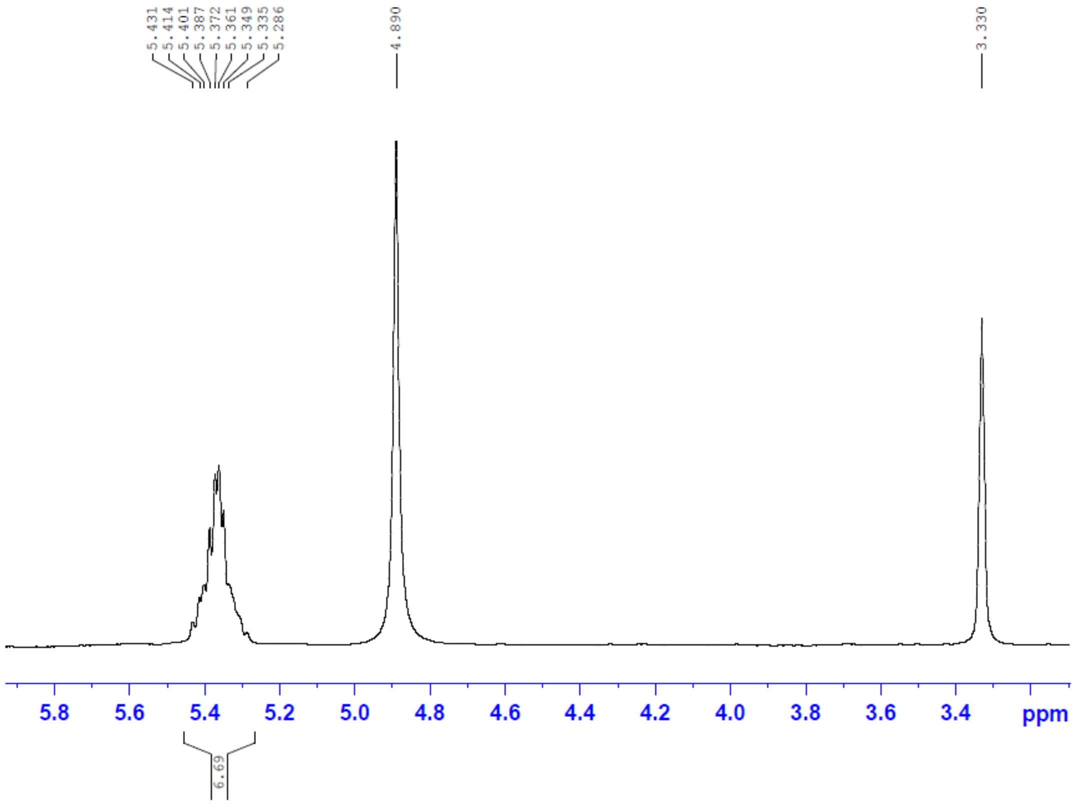

Supplementary Figure 2: ^{1}H NMR Spectral data (part-2) for the purified compound

EXCLI Journal 2026;25:50-67 – ISSN 1611-2156
Received: August 26, 2025, accepted: October 13, 2025, published: January 05, 2026

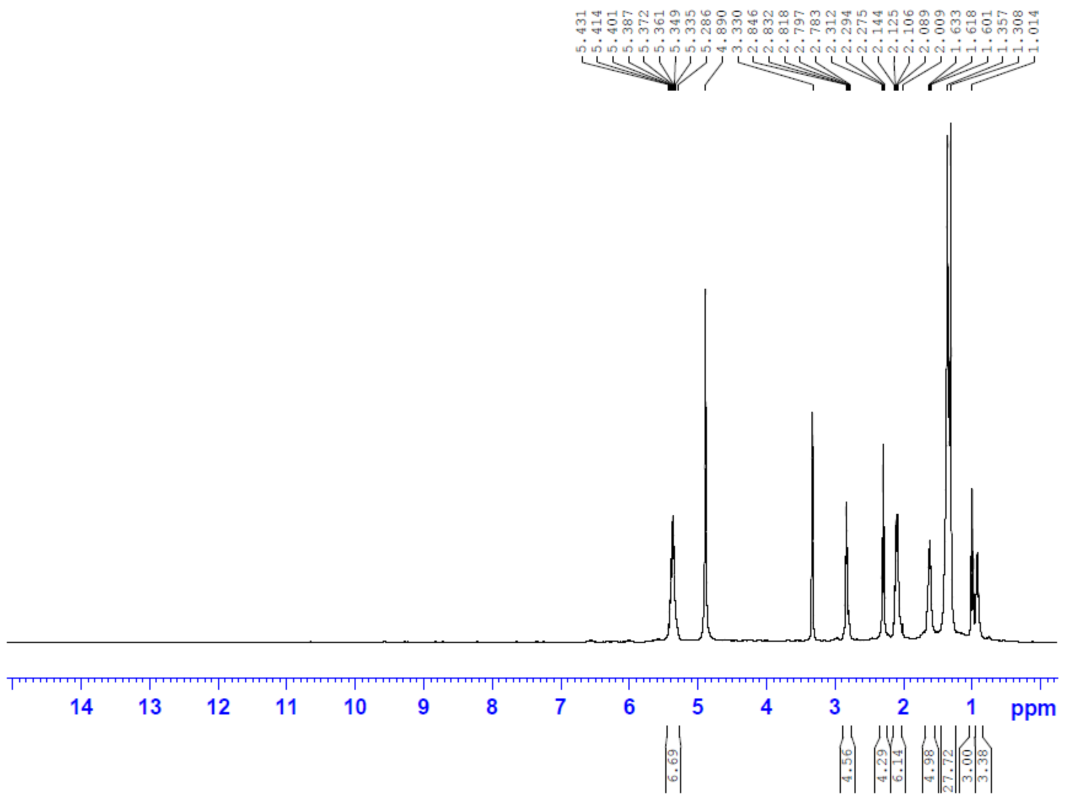

Supplementary Figure 3: ^{1}H NMR Spectral data (full) for the purified compound

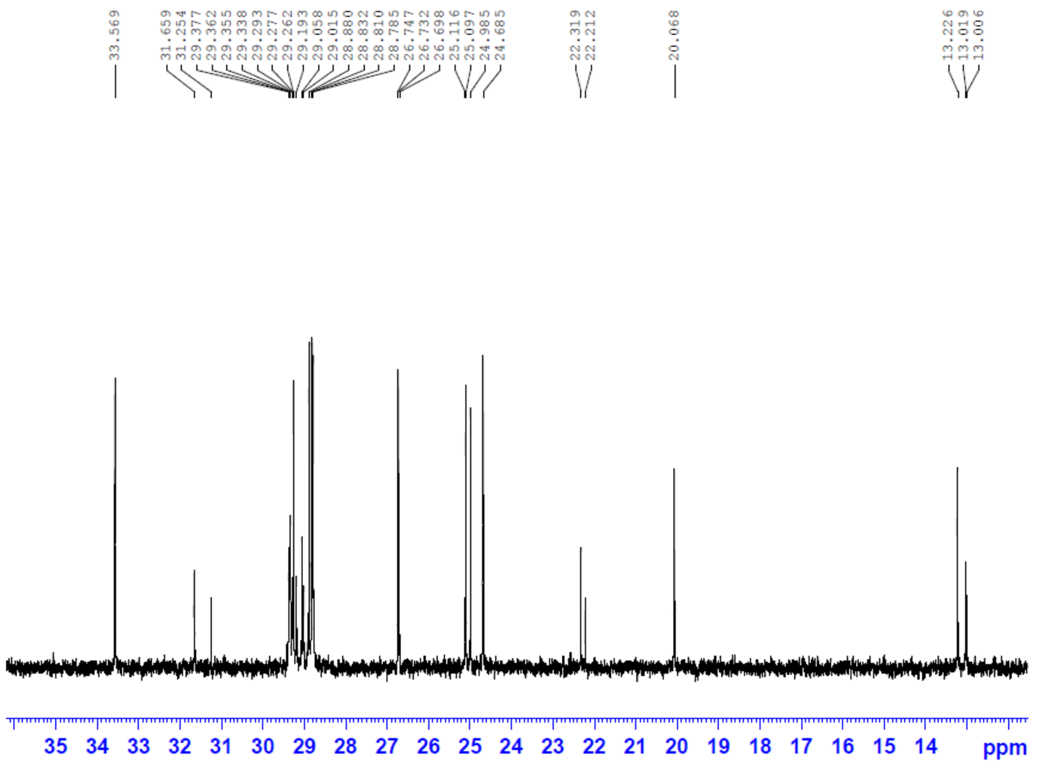

Supplementary Figure 4: ^{13}C NMR Spectral data (part-1) for the purified compound

EXCLI Journal 2026;25:50-67 – ISSN 1611-2156
Received: August 26, 2025, accepted: October 13, 2025, published: January 05, 2026

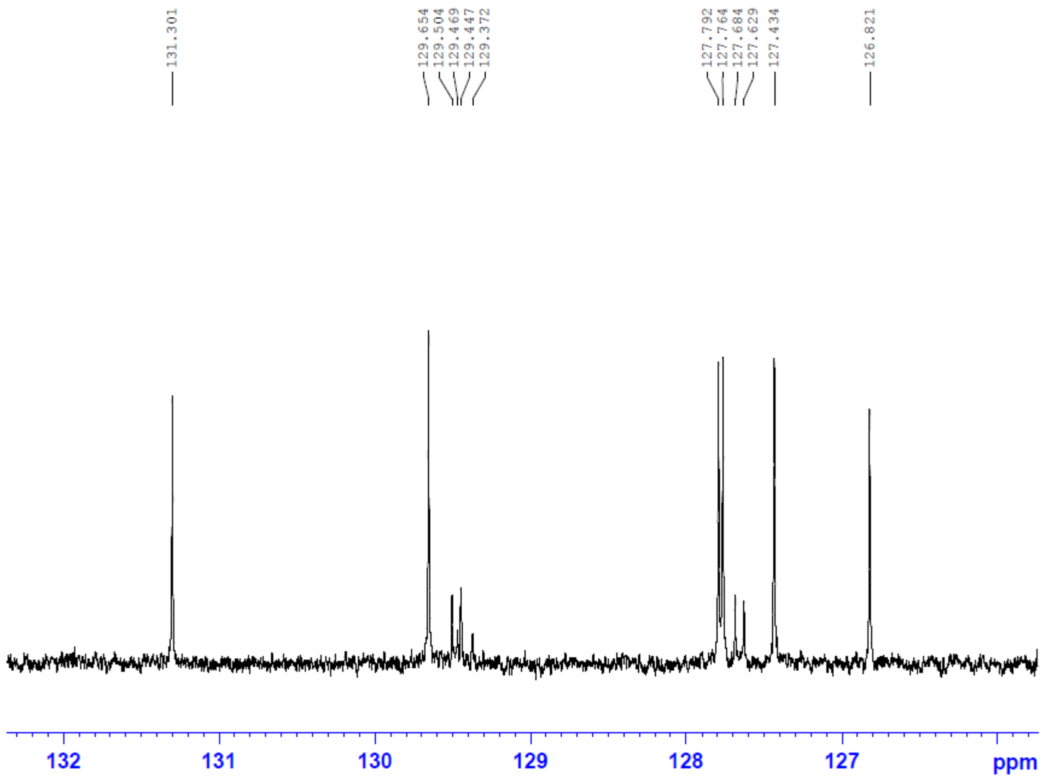

Supplementary Figure 5: ^{13}C NMR Spectral data (part-2) for the purified compound

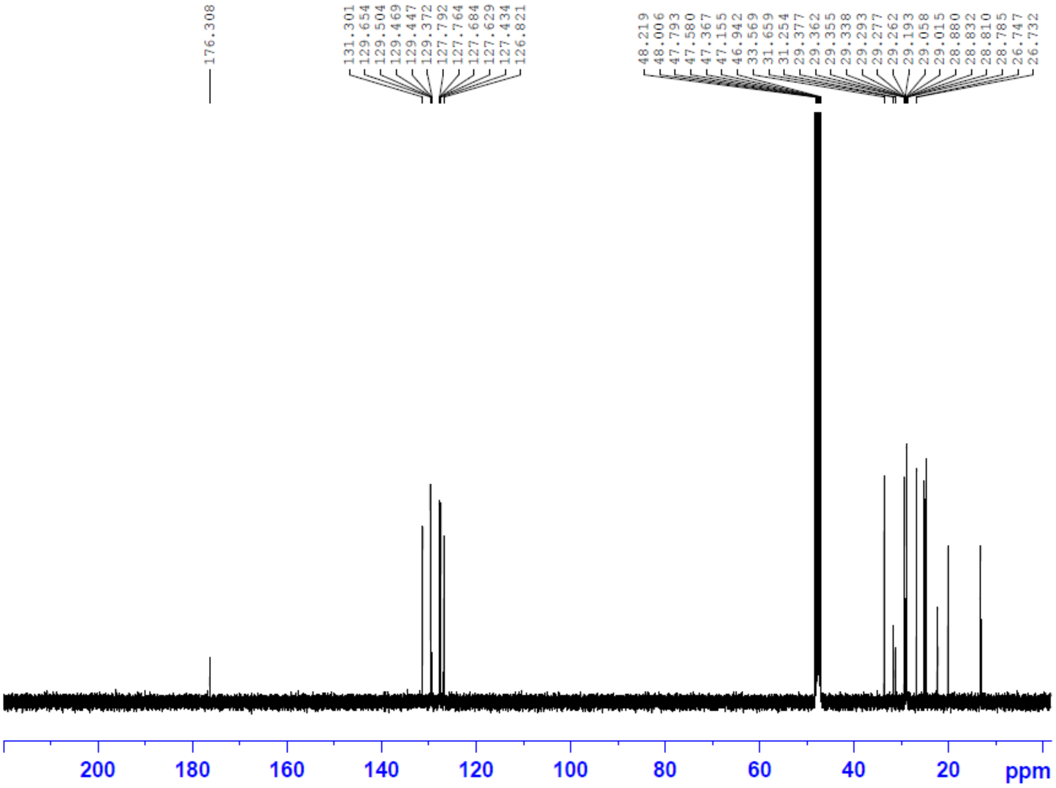

Supplementary Figure 6: ^{13}C NMR Spectral data (full) for the purified compound

EXCLI Journal 2026;25:50-67 – ISSN 1611-2156
Received: August 26, 2025, accepted: October 13, 2025, published: January 05, 2026

Supplementary Table 2: Unprocessed data for EAC viable cells. The number of viable EAC cells in the control and treated groups was obtained from independent animals (**Figure 2A**)

No. of mouse	Control group	ML (0.3 mg/kg)	ML (3.0 mg/kg)	Ifosfamide (0.4 mg/kg)
1	5.3 × 10^7	2.96 × 10^7	2.11 × 10^7	1.01 × 10^7
2	4.89 × 10^7	3.27 × 10^7	1.89 × 10^7	1.38 × 10^7
3	5.39× 10^7	3.55 × 10^7	1.21 × 10^7	1.23 × 10^7
4	6.88 × 10^7	3.58 × 10^7	2.29 × 10^7	1.12 × 10^7
5	6.43 × 10^7	3.12 × 10^7	2.03 × 10^7	1.17 × 10^7
6	5.78 × 10^7	3.3 × 10^7	1.91 × 10^7	1.18 × 10^7

Supplementary Table 3: Unprocessed data for cell growth inhibition (%) of the mouse in control and treatment groups (**Figure 2B**)

No. of mouse	ML (0.3 mg/kg)	ML (3.0 mg/kg)	Ifosfamide (0.4 mg/kg)
1	48.77	63.48	79.87
2	43.4	67.28	72.49
3	35.09	79.05	75.48
4	38.0408	60.37	76.08
5	46.002	64.86	76.28
6	42.88	67.11	75.48

Supplementary Table 4: Unprocessed data for survival time (days) of the mouse in control and treatment groups (**Figure 2C**)

No. of mouse	Control group	ML (0.3 mg/kg)	ML (3.0 mg/kg)
1	23	30	39
2	25	31	40
3	25	31	41
4	25	32	41
5	26	34	41
6	27	34	42

Supplementary Table 5: Unprocessed data for the % increase of lifespan of the mouse in control and treatment groups (**Figure 2D**)

No. of mouse	ML (0.3 mg/kg)	ML (3.0 mg/kg)
1	30.43478	69.56522
2	24	60
3	24	64
4	28	64
5	30.76923	57.69231
6	25.92593	55.55556

EXCLI Journal 2026;25:50-67 – ISSN 1611-2156
Received: August 26, 2025, accepted: October 13, 2025, published: January 05, 2026

Supplementary Table 6: Unprocessed data for the tumor weight/burden in grams of the mouse in control and treatment groups (**Figure 3A**)

Days	Control						ML (0.3 mg/kg)						ML (3.0 mg/kg)					
Mouse No.	1	2	3	4	5	6	1	2	3	4	5	6	1	2	3	4	5	6
0	0	0	0	0	0	0	0	0	0	0	0	0	0	0	0	0	0	0
2	0	1	1	1	0	1	0	0	0	1	0	1	1	0	1	0	1	1
4	2	2	2	3	2	3	1	1	1	1	1	2	1	1	1	1	1	1
6	3	4	4	5	3	4	2	1	2	2	2	2	1	1	1	1	1	1
8	4	5	5	6	5	6	3	2	3	2	3	4	1	1	2	0	1	2
10	6	5	6	8	8	8	2	3	3	4	3	2	0	2	1	2	1	2
12	8	8	9	8	9	9	3	3	3	5	4	2	2	1	2	2	2	2
14	10	10	10	10	10	11	4	5	6	4	5	4	3	2	2	1	2	2
16	10	12	12	12	12	12	6	5	6	5	5	5	3	3	2	2	3	3
18	12	13	13	14	12	14	6	5	7	5	6	5	3	3	2	3	3	3
20	14	14	14	16	14	16	8	7	8	6	6	5	4	4	3	4	4	4

Supplementary Table 7: Unprocessed data for RBC cells/ mL blood of the mouse in control and treatment groups (**Figure 3B**)

No. of mouse	Control group	ML (0.3 mg/kg)	ML (3.0 mg/kg)	Normal
1	5.12×10^9	5.8×10^9	6.25×10^9	7.05×10^9
2	5.64×10^9	5.78×10^9	6.51×10^9	7.09×10^9
3	5.79×10^9	6.13×10^9	7.44×10^9	7.89×10^9
4	4.1×10^9	6.8×10^9	6.62×10^9	7.67×10^9
5	4.62×10^9	5.92×10^9	7.03×10^9	6.9×10^9
6	4.9×10^9	5.98×10^9	6.38×10^9	6.9×10^9

Supplementary Table 8: Unprocessed data for WBC cells/ mL of blood of the mouse in control and treatment groups (**Figure 3C**)

No. of mouse	Control group	ML (0.3 mg/kg)	ML (3.0 mg/kg)	Normal
1	5800000	4000000	3300000	900000
2	6800000	4600000	1900000	900000
3	7200000	4500000	2800000	900000
4	7600000	5200000	3600000	900000
5	7800000	4900000	3100000	900000
6	6500000	5500000	2400000	100000

Supplementary Table 9: Unprocessed data for Hb gm/ dL of blood of the mouse in control and treatment groups (**Figure 3D**)

No. of mouse	Control group	ML (0.3 mg/kg)	ML (3.0 mg/kg)	Normal
1	4.6	5	5.9	7.9
2	4.2	4.8	6.1	8.1
3	4.9	6	6.8	7.5
4	5	5.8	7.2	7.4
5	5.1	5.2	7.4	7.8
6	4.8	5.3	6.7	7.6

EXCLI Journal 2026;25:50-67 – ISSN 1611-2156
Received: August 26, 2025, accepted: October 13, 2025, published: January 05, 2026

Supplementary Table 10: Unprocessed data for gene expression analysis (**Figure 6**). Data are shown as technical triplicates from independent experimental animals.

No. of mouse	p53	Bax	Caspase 3	Caspase 9	Bcl2
1	1.605	1.369	0.986	1.247	0.5474
	1.506	1.2935	1.547	1.27	0.4751
	1.574	1.354	1.314	1.26	0.7451
2	1.593	1.394	1.086	1.215	0.4374
	1.562	1.2853	1.557	1.212	0.5711
	1.541	1.514	1.283	1.124	0.6515
3	1.744	1.3131	1.714	1.361	0.6513
	1.483	1.059	1.143	1.3151	0.5344
	1.734	1.5842	1.184	1.3124	0.4972
4	1.578	1.333	1.062	1.223	0.4572
	1.6471	1.225	1.5571	1.2271	0.4454
	1.4752	1.3345	1.334	1.2361	0.5415
5	1.3974	1.2343	1.181	1.225	0.4443
	1.2872	1.3255	1.251	1.222	0.556
	1.8921	1.5424	1.2235	1.2341	0.564
6	1.499	1.3135	1.375	1.231	0.6554
	1.8324	1.251	1.2134	1.235	0.5541
	1.732	1.3545	1.248	1.2245	0.4427
